# Antiviral Activity of an Ethanolic Extract of *Cinnamomum cassia* and Cinnamaldehyde Against the Herpesviruses HSV-1 and VZV

**DOI:** 10.3390/cells15191829

**Published:** 2026-10-08

**Authors:** Baraa Alaasam, Yomna Alnabari, Mahmoud Huleihel

**Affiliations:** Department of Microbiology, Immunology and Genetics, Faculty of Health Sciences, Ben-Gurion University of the Negev, Beer-Sheva 8410501, Israel

**Keywords:** *Cinnamomum cassia*, cinnamaldehyde, herpesviruses, antiviral activity, natural products

## Abstract

Herpes simplex virus type 1 (HSV-1) and varicella–zoster virus (VZV) are clinically important alphaherpesviruses that establish lifelong latent infections and remain significant causes of morbidity despite the availability of antiviral drugs. The emergence of drug resistance and the inability of current therapies to eliminate latent virus reservoirs underscore the need for novel antiviral agents with alternative mechanisms of action. In the present study, we evaluated the antiviral activity of an ethanolic extract prepared from commercial cinnamon bark marketed as *Cinnamomum cassia* and cinnamaldehyde, a well-known constituent of cinnamon tested here as a purified compound, against HSV-1 and VZV in vitro. Antiviral activity was assessed using plaque reduction assays, time-of-addition experiments, and quantitative real-time PCR for viral DNA accumulation and specific viral immediate-early transcript levels. Both the *C. cassia* extract and cinnamaldehyde inhibited HSV-1 and VZV replication in a dose-dependent manner while exhibiting relatively low cytotoxicity. Nonlinear dose–response analysis yielded IC_50_ values of 3.44 and 2.80 µg/mL for the *C. cassia* extract against HSV-1 and VZV, respectively, and 0.961 and 1.50 µg/mL for cinnamaldehyde. Time-of-addition experiments demonstrated that antiviral activity was greatest when treatment was maintained throughout infection or initiated after viral entry, whereas pretreatment of cells or direct incubation with virus particles produced minimal effects. Both agents markedly reduced intracellular production of infectious progeny viruses and decreased viral DNA accumulation and the abundance of the specific immediate-early transcripts examined. These findings support a predominantly post-entry antiviral effect of both materials, rather than a primary effect on viral attachment or direct virion inactivation. Collectively, these results support further investigation of these agents as promising natural inhibitors of HSV and VZV infections.

## 1. Introduction

Herpesviruses are a large family of enveloped double-stranded DNA viruses that establish lifelong infections in humans, characterized by latency and periodic reactivation. Among them, herpes simplex virus type 1 (HSV-1) and type 2 (HSV-2), as well as varicella–zoster virus (VZV), are of major clinical importance. HSV-1 and HSV-2 are responsible for oral and genital lesions, keratitis, encephalitis, and severe complications in immunocompromised individuals, whereas VZV causes primary infection manifesting as varicella (chickenpox) and may later reactivate as herpes zoster (shingles) [1,2]. The global burden of these infections remains substantial, particularly in aging and immunocompromised populations, where reactivation can lead to severe neurological and systemic complications [2,3].

A hallmark of alphaherpesviruses, including HSV and VZV, is their ability to establish latency in sensory neurons and reactivate under conditions such as stress or immune suppression. In the case of VZV, reactivation is often associated with postherpetic neuralgia, a debilitating chronic pain condition that significantly affects quality of life [3,4]. Despite the availability of vaccines against VZV, breakthrough infections and reactivation still occur, underscoring the need for effective antiviral therapies targeting both primary infection and reactivation phases [4].

Current antiviral therapy for herpes simplex virus (HSV) and varicella–zoster virus (VZV) infections relies predominantly on nucleoside and nucleotide analogs, including acyclovir, valacyclovir, famciclovir, and related agents, which interfere with viral DNA synthesis. Although these drugs are generally effective in reducing viral replication and disease severity, they do not eliminate latent viral reservoirs, and their therapeutic efficacy may be compromised by delayed administration, incomplete viral suppression, and the emergence of drug-resistant variants, particularly in immunocompromised patients [4,5,6,7]. Recent advances in HSV antiviral development have therefore focused on agents with mechanisms distinct from those of conventional DNA polymerase inhibitors, including helicase–primase inhibitors and other compounds targeting alternative stages of the viral replication cycle. These developments underscore the ongoing need to identify antiviral agents with novel or multitarget mechanisms of action that may complement or overcome the limitations of existing therapies [8].

Natural products continue to represent an important source of structurally diverse antiviral compounds and potential lead molecules for the development of new therapeutic strategies. Recent studies and reviews have highlighted the ability of plant-, marine-, and microorganism-derived compounds to interfere with multiple stages of herpesvirus infection, including viral attachment and entry, intracellular genome replication, viral gene expression, protein synthesis, virion assembly, and host signaling pathways [9,10,11,12,13,14,15]. Such multitarget activity is particularly attractive in the context of herpesvirus infections because it may provide opportunities to develop agents with mechanisms distinct from conventional nucleoside analogues and potentially reduce selective pressure on a single viral target. Recent experimental studies have further demonstrated that crude botanical extracts and their major isolated constituents can selectively inhibit HSV replication, supporting continued efforts to identify and characterize bioactive antiviral compounds from natural sources [11,16].

*Cinnamomum cassia* (Chinese cinnamon) is a medicinal plant widely used in traditional medicine and known for its antimicrobial, antioxidant, anti-inflammatory, and antiviral properties [16,17]. *C. cassia* contains a variety of bioactive compounds, and cinnamaldehyde is widely recognized as a characteristic constituent of cinnamon and has been associated with many of its biological effects [18]. However, the composition and cinnamaldehyde content of the ethanolic extract tested in the present study were not determined. Cinnamaldehyde, a phenylpropanoid compound and a major component of cinnamon oil, is used in traditional Chinese medicine and has been extensively studied for its antimicrobial and antiviral activities against a broad range of pathogens, including enveloped viruses [17,19,20,21]. However, the antiviral properties of cinnamon-derived preparations may depend substantially on the botanical species, extraction method, chemical composition, formulation, and virus examined.

Several studies have demonstrated that cinnamaldehyde exhibits antiviral activity through multiple mechanisms, including disruption of viral envelope integrity, inhibition of viral protein synthesis, and modulation of host cellular pathways [20,21,22]. In vitro studies have shown that *cinnamaldehyde* and related phenolic compounds can inhibit viral replication and reduce viral infectivity. Notably, essential oils and extracts derived from cinnamon species have demonstrated virucidal activity against HSV, particularly by interfering with early stages of viral infection [22]. Given that both HSV and VZV are enveloped viruses with similar entry and replication strategies, these findings suggest that cinnamaldehyde and cinnamon-derived extracts may also exhibit activity against VZV, although this remains underexplored.

Recent investigations have further supported the antiviral potential of *cinnamaldehyde* and related compounds, demonstrating their ability to interact with viral or host targets involved in infection and replication [11,15,16,17,18,19,20,21,22,23,24,25]. Cinnamaldehyde has been reported to exhibit antiviral activity against several enveloped and non-enveloped viruses through mechanisms that may involve both direct effects on viral replication and modulation of host–cell processes [18]. In particular, cinnamaldehyde and preparations derived from *Cinnamomum* species have demonstrated inhibitory activity against HSV-1 in vitro. Zhou et al. [26] reported that cinnamaldehyde, as well as several *Cinnamomi ramulus* extracts, inhibited HSV-1 replication, supporting the presence of antiviral activity associated with this compound and cinnamon-derived preparations. In addition, studies in other viral systems have shown that cinnamaldehyde can inhibit viral replication when administered after infection and may interfere with viral protein synthesis, suggesting that its antiviral activity is not necessarily restricted to direct virucidal effects or inhibition of viral entry [27].

Cinnamaldehyde may also influence host–cell pathways that are relevant to productive viral infection. Experimental studies have demonstrated that cinnamaldehyde and related cinnamaldehyde derivatives can inhibit NF-κB activation and modulate MAPK-associated signaling pathways. These observations are of potential relevance to HSV-1 infection because activation of NF-κB has been associated with efficient HSV-1 replication and the regulation of cellular survival responses in infected cells [28,29,30]. Thus, although the precise molecular targets responsible for the anti-HSV-1 activity of cinnamaldehyde remain incompletely defined, the available literature supports the possibility that its antiviral effects may involve both interference with viral replication processes and modulation of host pathways required for efficient infection.

These findings highlight the potential of cinnamaldehyde as a multitarget antiviral agent. However, the antiviral activity of *Cinnamomum cassia* ethanolic extract and cinnamaldehyde, tested separately as a purified compound, against herpesviruses, particularly in a comparative context between HSV and VZV, remains incompletely characterized. Despite growing evidence supporting the broader antiviral potential of cinnamaldehyde and other cinnamon-derived constituents, their activity against alpha herpesviruses remains insufficiently characterized. In particular, relatively little is known about whether *C. cassia* ethanolic extract and cinnamaldehyde can inhibit both HSV-1 and VZV through comparable mechanisms or whether their activity primarily affects viral entry, direct virion infectivity, or intracellular stages of the replication cycle. This knowledge gap provides a rationale for directly comparing the antiviral activity and timing-dependent effects of *C. cassia* extract and cinnamaldehyde against these two clinically important alpha herpesviruses.

Therefore, in the present study, we evaluate the antiviral activity of an ethanolic extract of *Cinnamomum cassia* and cinnamaldehyde against HSV-1 and VZV. By investigating their effects on viral infectivity and replication, this work aims to advance the development of novel plant-based antiviral agents with broader therapeutic applicability.

## 2. Materials and Methods

### 2.1. Preparation of Cinnamomum Ethanolic Extract

Commercially obtained dried cinnamon bark marketed as *Cinnamomum cassia* was used for extract preparation. The material was obtained from a local commercial source and was not supplied with formal botanical certification. Taxonomic authentication and a herbarium voucher specimen were therefore not available, and the exact botanical identity of the commercial plant material could not be independently verified. Two grams of finely ground dried bark were extracted with 95% ethanol at 4 °C under continuous agitation for 48 h, centrifuged at 2000 rpm for 10 min, and the supernatant was collected. The collected supernatant was dried by evaporation and then dissolved in 95% ethanol to obtain a 10 mg/mL stock solution. The extract was diluted with medium containing 2% newborn calf serum (NBCS) to the required concentrations.

Cinnamaldehyde was purchased from Sigma-Aldrich, Rehovot, Israel (Cat. No. W228613; >95% purity).

### 2.2. Cells and Viruses

African green monkey kidney (Vero 76) cells were purchased from the American Type Culture Collection (ATCC), Rockville, MD, USA and grown [31]. Vero cells are highly permissive to herpesvirus infection and represent a well-established model for antiviral screening.

HSV-1 was obtained from ATCC (VR-735), and VZV was obtained from the virology laboratory at Soroka University Medical Center, Beer-Sheva, Israel.

### 2.3. Cytotoxicity Examination

Vero cells were plated at 4 × 10^5^ cells/well in 24-well culture plates and grown in an RPMI medium with 10% fetal calf serum, 1% glutamine, and 50 µg/mL antibiotic mixture (penicillin and streptomycin), and incubated at 37 °C in humidified air containing 5% CO_2_. All the supplements for the Vero cultivation were purchased from Biological Industries (Beit-Haemek, Israel). The cells were treated with various doses of either *Cinnamomum ethanolic extract*, Cinnamaldehyde, or *Acyclovir* (*ACV*), and their cytotoxicity was tested by two methods during a three-day period: (1) daily morphological observations using an inverted optical microscope; and (2) the (sodium 3′-[1-(phenylaminocarbonyl)-3,4-tetrazolium]-bis (4-methoxy6-nitro) benzene sulfonic acid hydrate) colorimetric assay (XTT Cell Proliferation Assay Kit, Sigma-Aldrich) for the quantification of cellular proliferation, viability, and cytotoxicity, as described previously [32]. Untreated cells were used as controls, and the percentage of cell viability was calculated as the absorbance of treated wells divided by the absorbance of untreated control wells, multiplied by 100.

### 2.4. Viral Adsorption

Vero cell monolayers were inoculated with HSV-1 or VZV at 1 PFU/cell in RPMI medium containing 2% NBCS and incubated at 37 °C for a 2 h viral adsorption period. Following adsorption, the inoculum was removed and subsequent procedures were performed as described below. Infection development was evaluated by plaque assay as previously described [31].

### 2.5. Plaque Assay

Following the 2 h viral adsorption period, unadsorbed virus particles were removed and the cells were covered with medium containing carboxymethylcellulose (CMC), in the presence or absence of the treatment solution, and incubated for 2 days. Then the CMC overlay was removed, and the cell monolayers were fixed with 10% formaldehyde in saline, stained with crystal violet, and the plaques counted. The antiviral effect (%) was calculated as the number of plaques in untreated cells minus the number of plaques in treated cells, divided by the number of plaques in untreated cells, and multiplied by 100.

### 2.6. Time-of-Addition Assay

Vero cells were treated with 100 µg/mL of either *C. cassia* extract or cinnamaldehyde at different stages relative to infection, as follows: (1) Cell pretreatment: cells were pretreated with the tested material for 2 h before infection and then washed twice with 0.9% NaCl solution. (2) During adsorption: cells were treated with the tested material during the 2 h virus adsorption period and then washed twice with 0.9% NaCl solution. (3) Post-entry: after the 2 h adsorption period, cells were incubated with the tested material throughout the subsequent 2-day post-infection period. (4) Adsorption + post-entry: cells were treated during the 2 h adsorption period, and treatment was maintained throughout the subsequent 2-day post-infection period. (5) Virus-compound preincubation: to assess a possible direct effect of the tested material on viral particle infectivity, viral particles were pre-incubated with the tested material at room temperature for 1 h. The mixtures were then diluted 10^4^-fold with fresh medium to minimize the test-material concentration during infection, and the diluted mixtures were used to infect cells.

### 2.7. Assessment of Intracellular Virus Production

Cells were infected with HSV-1 or VZV for 2 h without treatment. The medium was then replaced with fresh medium with or without the tested product, and cells were harvested at 20 or 35 h post-infection using trypsin. After centrifugation (1500 rpm, 5 min) and three saline washes, cell pellets were resuspended in 100 µL saline, lysed by freeze–thaw cycles, and centrifuged again to remove debris. The resulting virus-containing supernatant was used to infect cell monolayers.

### 2.8. DNA Analysis

For viral DNA analysis, infected cells were harvested at 24 h post-infection. Viral DNA extraction was performed using the Genomic DNA Purification Kit (Thermo Fisher Scientific, Waltham, MA, USA) according to the manufacturer’s instructions. Viral DNA was quantified by SYBR Green-based real-time PCR using virus-specific primer sets. HSV-1 DNA was amplified using 5′-CGTACCTGCGGCTCGTGAAGT-3′ and 5′-AGCAGGGTGCTCGTGTATGGGC-3′, generating a 271 bp HSV-1-specific amplicon as previously described [33]. VZV DNA was amplified using 5′-CATTACCGCACCCAAAGTGAA-3′ and 5′-CTAACGCTTCCACCTCGGGT-3′, generating an 89 bp VZV-specific amplicon as previously described [34]. Cellular β-actin DNA was used as the endogenous reference for normalization using the primers 5′-GCACCCAGCACAATGAAGA-3′ and 5′-CGATCCACACGGAGTACTTG-3′. Relative viral DNA levels were calculated using the 2^−ΔΔCt^ method [35], where ΔCt was calculated as Ct(viral target) − Ct(β-actin). Infected untreated cells were used as the calibrator and assigned a relative viral DNA level of 100%. No absolute viral copy-number determination or standard curve was used.

### 2.9. RNA Analysis

For RNA analysis, HSV-1-infected cells were harvested at 4 h post-infection and VZV-infected cells at 14 h post-infection. Total RNA was extracted from HSV-1- or VZV-infected Vero cells using the GenElute Mammalian Total RNA Miniprep Kit (Merck, Darmstadt, Germany) according to the manufacturer’s instructions. RNA samples were treated with DNase to remove contaminating DNA prior to reverse transcription. RNA was reverse-transcribed to cDNA using the qScript cDNA Synthesis Kit (Quantabio, Beverly, MA, USA). Viral transcript levels were determined by SYBR Green-based quantitative real-time PCR. For VZV, the immediate-early ORF63 (IE63) transcript was amplified using the primers 5′-TCGGACGGGGAAGACTTTAT-3′ and 5′-CGTCTGGTTCACAAGAATCG-3′ [36]. For HSV-1, the ICP4/RS1 immediate-early (α) transcript was amplified using the primers 5′-CGGTGATGAAGGAGCTGCTGTTGC-3′ and 5′-CTGATCACGCGGCTGCTGTACA-3′ [37]. β-actin was used as the endogenous reference gene using the primers mentioned above. Relative viral transcript levels were calculated using the 2^−ΔΔCt^ method [35], with infected untreated cells serving as the calibrator and assigned a relative expression level of 100%.

### 2.10. Statistical Analyses

**Figure 4:**
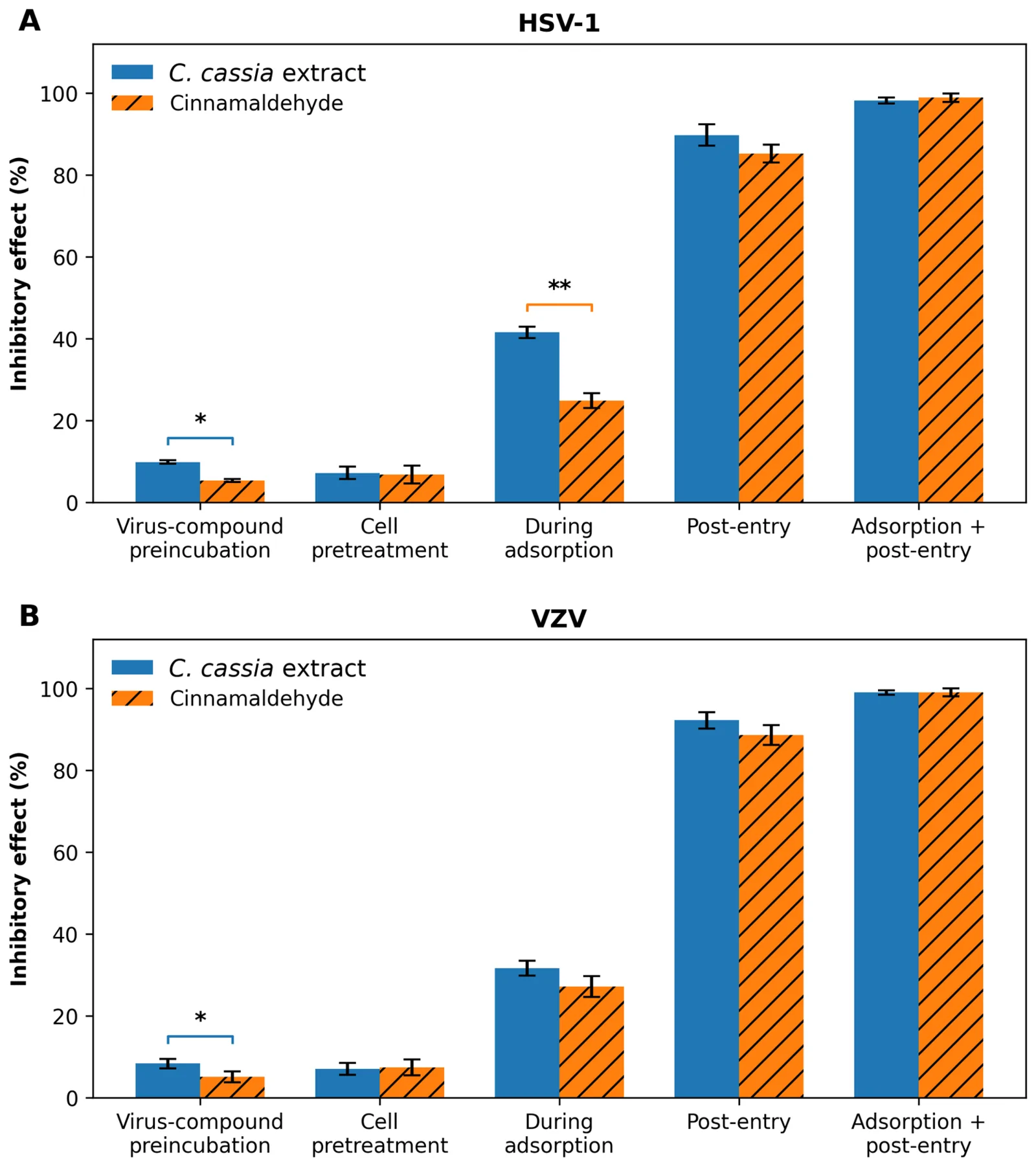
*Time-of-addition assay of C. cassia extract and cinnamaldehyde against HSV-1 and VZV*. Vero cells were infected with HSV-1 (**A**) or VZV (**B**) at an MOI of 1 and exposed to 100 μg/mL *C. cassia* extract or cinnamaldehyde under the indicated conditions: cell pretreatment, in which cells were exposed to the tested material for 2 h before viral inoculation and subsequently washed; during adsorption, in which treatment was present only during the 2 h viral adsorption period; post-entry, in which treatment was added following the 2 h adsorption period and maintained thereafter; and adsorption + post-entry, in which treatment was present during adsorption and maintained throughout the subsequent experimental period. For virus-compound preincubation, 10^5^ PFU of HSV-1 or VZV were incubated with the tested material at room temperature for 1 h, diluted 10^4^-fold, and then used to inoculate Vero cell monolayers. Antiviral activity was determined by plaque assay and expressed as inhibition relative to untreated infected controls. Each condition was evaluated in three independent experiments performed on different days, each in technical triplicate. Technical triplicates were averaged within each independent experiment, and bars represent mean ± SD (n = 3). Data were analyzed by two-way repeated-measures ANOVA followed by Šídák’s multiple-comparisons test. Significant pairwise comparisons between *C. cassia* extract and cinnamaldehyde within each treatment condition are indicated: * *p* < 0.05, ** *p* < 0.01.

**Figure 5:**
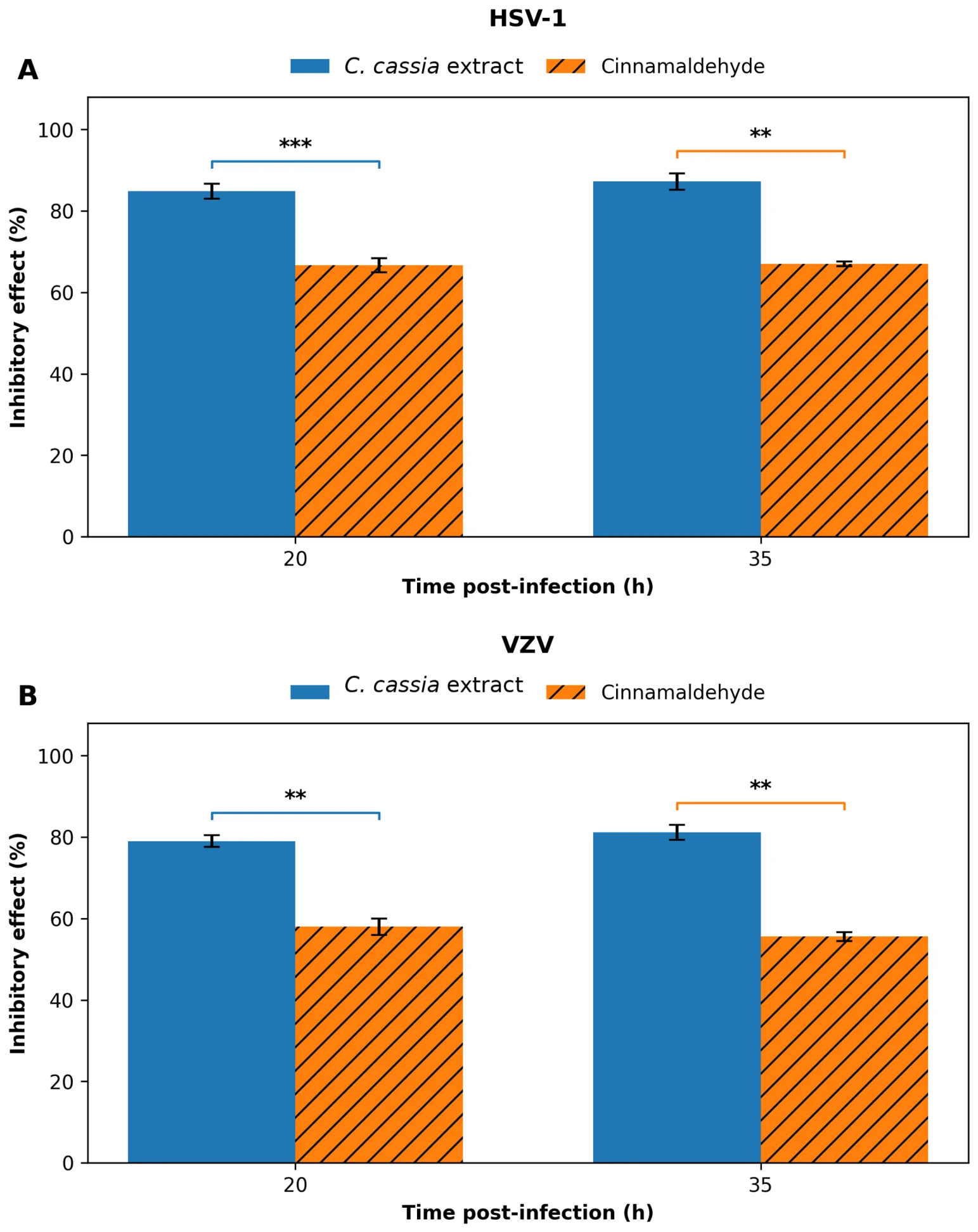
*Effect of C. cassia extract and cinnamaldehyde on infectious progeny virus production.* Vero cell monolayers were infected with HSV-1 (**A**) or VZV (**B**) at an MOI of 1. Following the 2 h viral adsorption period, the inoculum was removed and replaced with fresh medium containing 100 μg/mL *C. cassia* extract or cinnamaldehyde. At 20 and 35 h post-infection, intracellular virus was released by freeze–thaw cycles and infectivity was determined by plaque assay on fresh Vero cell monolayers. Inhibition of progeny virus production was calculated relative to untreated infected controls. Each condition was evaluated in three independent experiments performed on different days, each in technical triplicate; technical triplicates were averaged within each independent experiment and bars represent mean ± SD (n = 3). Data were analyzed by two-way repeated-measures ANOVA followed by Šídák’s multiple-comparisons test. Significant pairwise differences between *C. cassia* extract and cinnamaldehyde at each time point are indicated: ** *p* < 0.01, *** *p* < 0.001.

**Figure 6:**
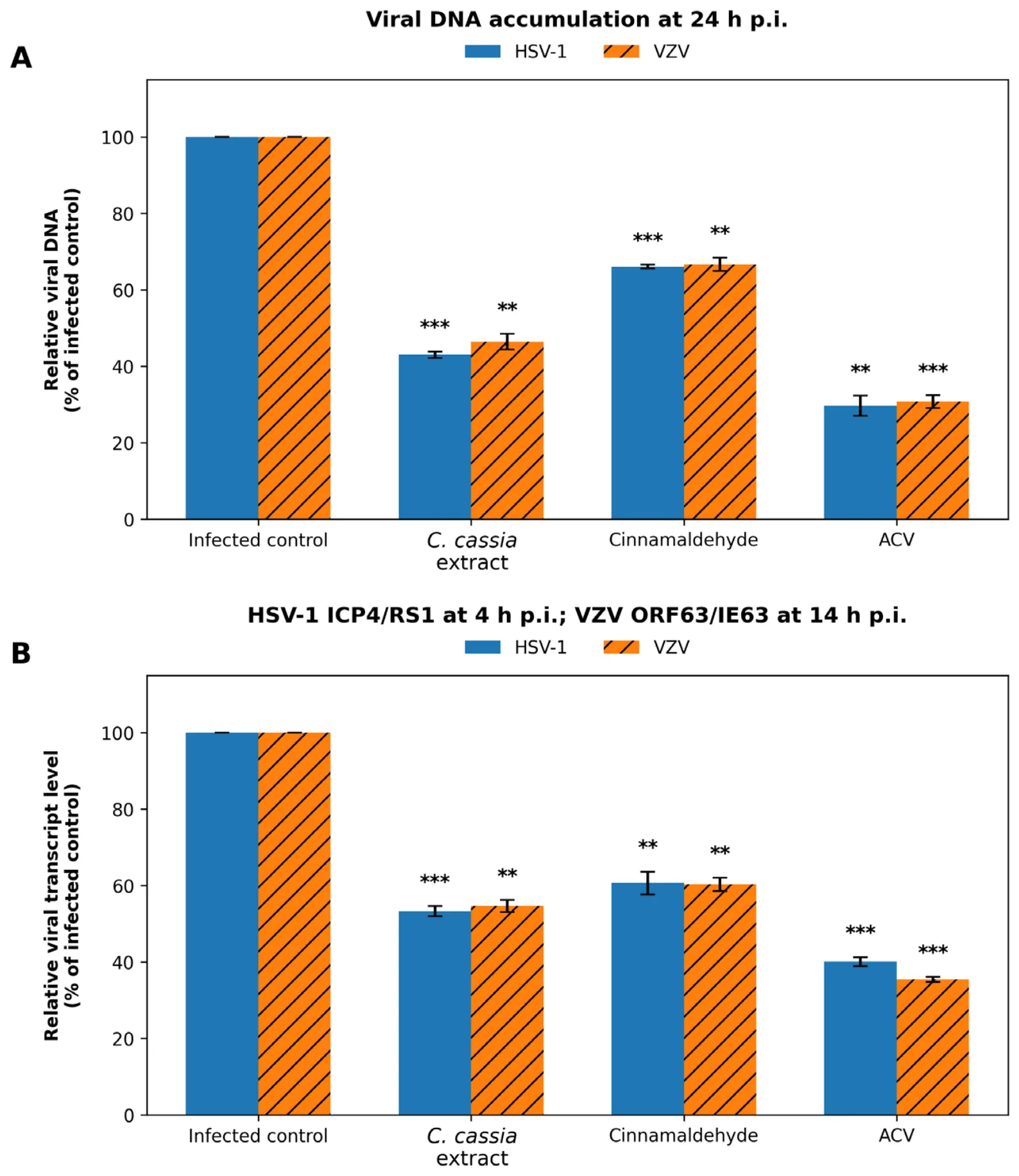
*Effect of C. cassia extract and cinnamaldehyde on viral DNA accumulation and specific viral immediate-early transcript levels*. (**A**) Viral DNA accumulation was determined at 24 h post-infection. (**B**) The HSV-1 immediate-early ICP4/RS1 transcript was quantified at 4 h post-infection, whereas the VZV immediate-early ORF63/IE63 transcript was quantified at 14 h post-infection. Vero cells infected with HSV-1 or VZV were treated following the 2 h viral adsorption period with 100 µg/mL *C. cassia* ethanolic extract, 100 µg/mL cinnamaldehyde, or 10 µg/mL acyclovir (ACV). Viral DNA and transcript levels were quantified by real-time PCR, normalized to β-actin, and expressed relative to the corresponding untreated infected control, which was assigned a value of 100%. Each condition was evaluated in three independent experiments performed on different days, each in technical triplicate. Technical triplicates were averaged within each independent experiment, and bars represent mean ± SD (n = 3). Statistical significance for each treatment relative to the corresponding infected control was evaluated using two-sided one-sample *t*-tests against 100%, with Šídák correction for the three treatment comparisons within each virus and panel. ** *p* < 0.01; *** *p* < 0.001.

All experiments were independently repeated three times on different days, and each condition within each independent experiment was assayed in technical triplicate. Technical triplicates were averaged to obtain a single value for each independent experiment; therefore, n = 3 represents three independent experiments. Data are expressed as the mean ± SD of the three independent experiments. For concentration-response analyses, the technical triplicates within each independent experiment were averaged first, and the resulting values were analyzed by nonlinear least-squares regression using a normalized variable-slope sigmoidal (Hill) dose–response model. For the normalized curves, the lower and upper plateaus were constrained to 0% and 100%, respectively. The 50% cytotoxic concentration (CC_50_) and 50% inhibitory concentration (IC_50_) were obtained from the fitted curves, and goodness of fit was assessed using the coefficient of determination (R^2^). The selectivity index (SI) was calculated as CC_50_/IC_50_. For the time-of-addition and progeny-virus experiments shown in Figure 4 and Figure 5, technical triplicates were averaged within each independent experiment before statistical analysis. For each virus, data were analyzed by two-way repeated-measures ANOVA, with tested material and treatment timing (Figure 4) or sampling time (Figure 5) as within-experiment factors. Where appropriate, Šídák-adjusted pairwise comparisons were performed between *C. cassia* extract and cinnamaldehyde within each treatment condition or time point. A two-tailed adjusted *p*-value < 0.05 was considered statistically significant. Nonlinear regression and statistical analyses were performed using GraphPad Prism 7.02 (San Diego, CA, USA). For the relative qPCR data shown in Figure 6, technical triplicates were first averaged within each independent experiment. Because the untreated infected group served as the calibrator and was defined as 100%, treatment values from the three independent experiments were compared with the reference value of 100% using two-sided one-sample *t*-tests against 100%, with Šídák correction for the three treatment comparisons within each virus and panel. A two-tailed adjusted *p*-value < 0.05 was considered statistically significant.

## 3. Results

### 3.1. Cytotoxicity

**Figure 1:**
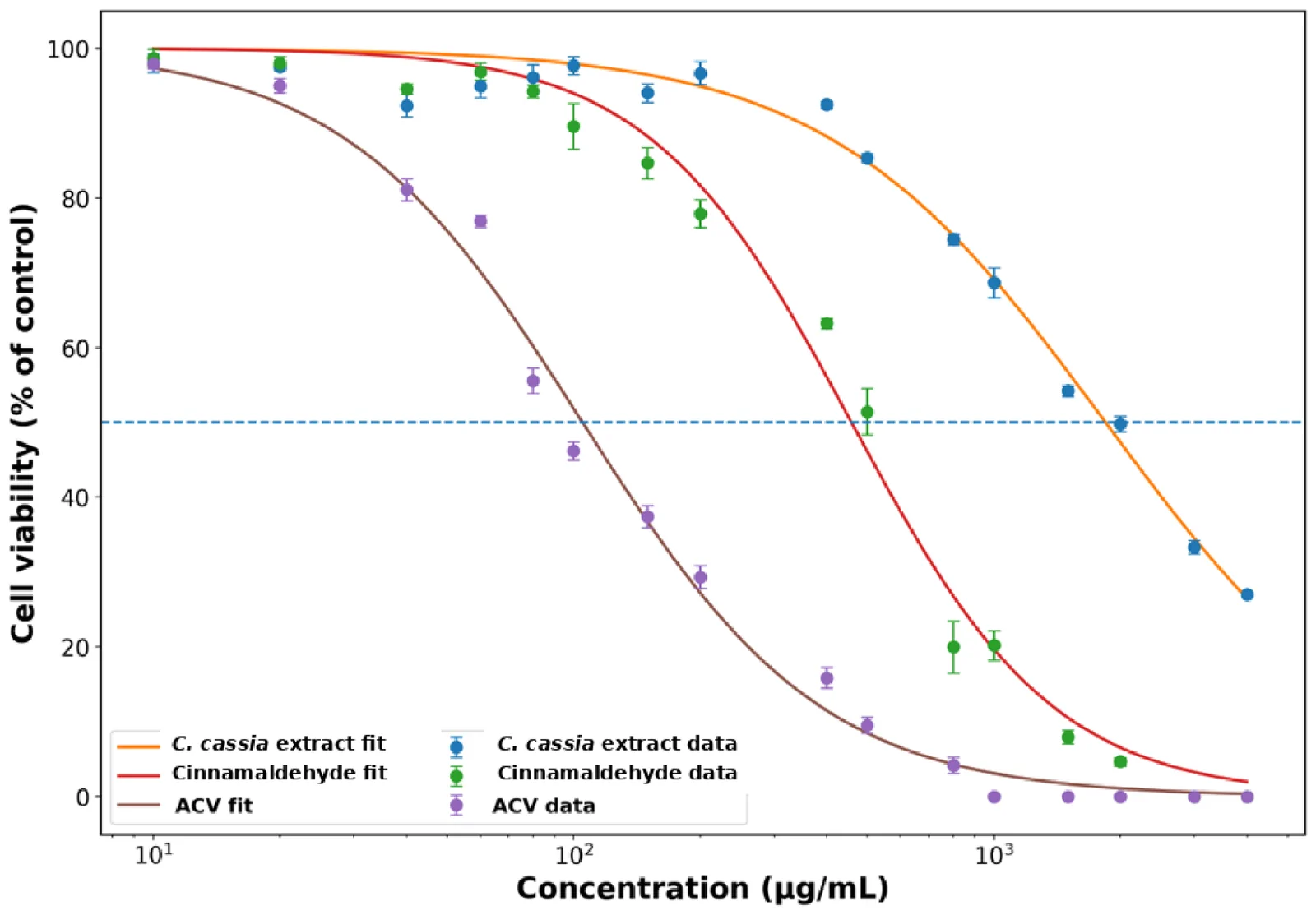
Cytotoxicity dose–response curves of *C. cassia* extract, cinnamaldehyde, and acyclovir (ACV) in Vero cells. Vero cells were treated with increasing concentrations of the indicated substances for 72 h, and cell viability was determined using the XTT assay. Points represent the mean ± SD of three independent experiments, each performed in triplicate, after averaging the technical triplicates within each experiment. Solid lines represent the best-fit nonlinear regression curves obtained using a normalized variable-slope sigmoidal model. The regression-derived CC_50_ values were 1843.7 µg/mL for *C. cassia* extract (R^2^ = 0.9864), 458.3 µg/mL for cinnamaldehyde (R^2^ = 0.9909), and 105.0 µg/mL for ACV (R^2^ = 0.9928). The dashed horizontal line indicates 50% cell viability.

Vero cell monolayers were treated with increasing concentrations of *C. cassia* bark ethanolic extract, cinnamaldehyde, or acyclovir (ACV) for 3 days, and cell viability was evaluated by daily morphological observations and the XTT assay as described in the Materials and Methods. Nonlinear regression analysis of the complete concentration-response datasets yielded CC_50_ values of 1843.7 µg/mL for *C. cassia* extract, 458.3 µg/mL for cinnamaldehyde, and 105.0 µg/mL for ACV. The corresponding goodness-of-fit values were R^2^ = 0.9864, 0.9909, and 0.9928, respectively (Figure 1).

### 3.2. Antiviral Activity of Cinnamomum Extract and Cinnamaldehyde Against Herpesviruses

**Figure 2:**
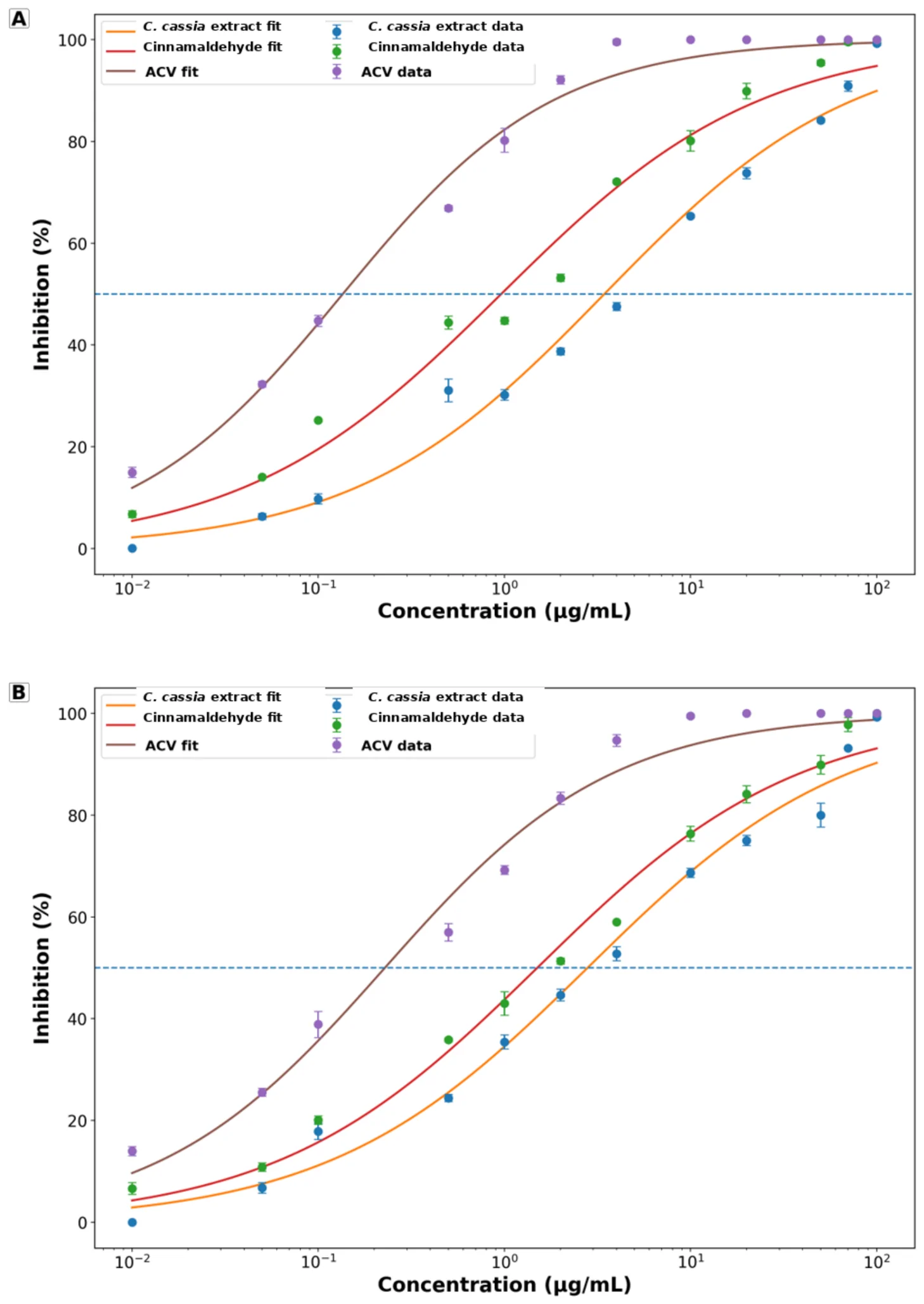
Nonlinear dose–response analysis of antiviral activity against HSV-1 and VZV. Vero cells were infected with HSV-1 (**A**) or VZV (**B**) at a multiplicity of infection (MOI) of 1 and treated with increasing concentrations of *C. cassia* extract, cinnamaldehyde, or ACV. Points represent the mean ± SD of three independent experiments, each performed in triplicate, after averaging the technical triplicates within each experiment. Solid lines represent the best-fit normalized variable-slope sigmoidal regression curves. For HSV-1, the IC_50_ values were 3.442 µg/mL for *C. cassia* extract (R^2^ = 0.9831), 0.961 µg/mL for cinnamaldehyde (R^2^ = 0.9810), and 0.135 µg/mL for ACV (R^2^ = 0.9878). For VZV, the IC_50_ values were 2.799 µg/mL for *C. cassia* extract (R^2^ = 0.9833), 1.503 µg/mL for cinnamaldehyde (R^2^ = 0.9871), and 0.228 µg/mL for ACV (R^2^ = 0.9830). The dashed horizontal line indicates 50% inhibition.

Vero cell monolayers were treated with various concentrations of either *Cinnamomum* extract or cinnamaldehyde at the time of infection with HSV-1 or VZV at a multiplicity of infection (MOI) of 1. Treatment was maintained throughout the experimental period, and antiviral activity was assessed using a plaque reduction assay. Both the *C. cassia* extract and cinnamaldehyde inhibited HSV-1 and VZV replication in a concentration-dependent manner. Nonlinear regression analysis of the complete dose–response data yielded an IC_50_ of 3.442 µg/mL for the *C. cassia* extract against HSV-1 (R^2^ = 0.9831) and 2.799 µg/mL against VZV (R^2^ = 0.9833). Cinnamaldehyde yielded IC_50_ values of 0.961 µg/mL against HSV-1 (R^2^ = 0.9810) and 1.503 µg/mL against VZV (R^2^ = 0.9871). For the reference drug ACV, the IC_50_ values were 0.135 µg/mL against HSV-1 (R^2^ = 0.9878) and 0.228 µg/mL against VZV (R^2^ = 0.9830) (Figure 2).

**Figure 3:**
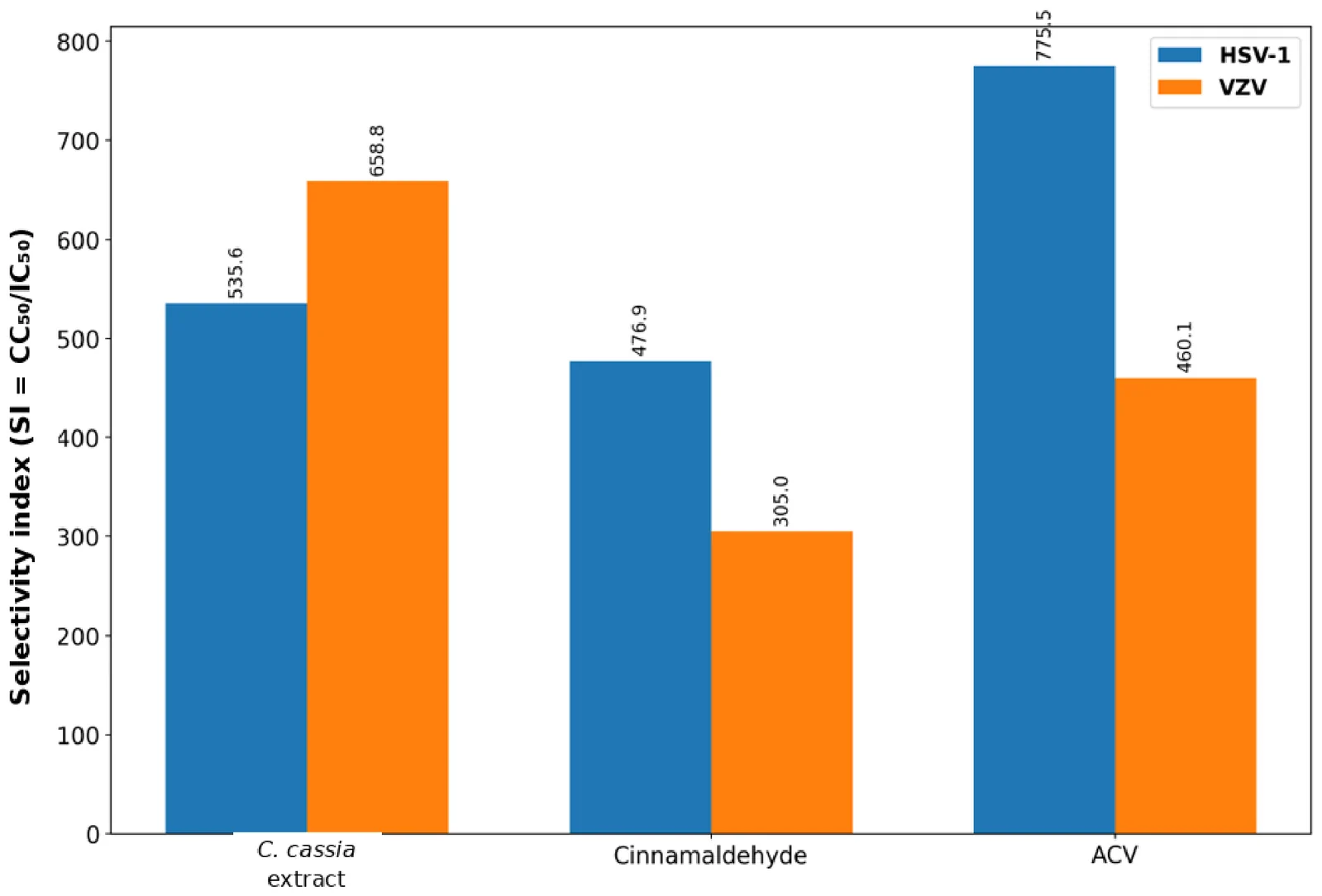
Selectivity index (SI) of *C. cassia* extract, cinnamaldehyde, and acyclovir (ACV) against HSV-1 and VZV. SI values were calculated from the nonlinear regression-derived parameters as CC_50_/IC_50_. The resulting SI values for HSV-1 were 535.6, 476.9, and 775.5 for the extract, cinnamaldehyde, and ACV, respectively; the corresponding values for VZV were 658.8, 305.0, and 460.1.

Using the regression-derived CC_50_ and IC_50_ values, the selectivity indices (SI = CC_50_/IC_50_) were 535.6 and 658.8 for the *C. cassia* extract against HSV-1 and VZV, respectively; 476.9 and 305.0 for cinnamaldehyde; and 775.5 and 460.1 for ACV (Figure 3). Thus, the extract and cinnamaldehyde showed similar selectivity against HSV-1, whereas the extract showed a markedly higher selectivity index than cinnamaldehyde against VZV.

### 3.3. Time-of-Addition Assay

To characterize the timing dependence of the antiviral activity of the tested extract and cinnamaldehyde, infected cells were exposed to 100 µg/mL of these substances at different stages of herpesvirus infection. The strongest antiviral activity was observed when treatment was initiated simultaneously with viral inoculation and maintained throughout the infection period, resulting in nearly complete suppression of viral infection (Figure 4). Administration of either the extract or cinnamaldehyde exclusively after viral entry also produced substantial inhibition, reducing viral infection by approximately 85–90%. In contrast, treatment limited to the adsorption phase yielded only moderate antiviral activity, with the extract and cinnamaldehyde reducing infection by approximately 40% and 25%, respectively. Pretreatment of host cells or direct incubation of viral particles with either substance produced only minimal reductions in infectivity.

Time-of-addition analysis demonstrated a highly significant effect of treatment timing for both HSV-1 and VZV (two-way repeated-measures ANOVA, *p* < 0.0001 for both viruses). For HSV-1, there was also a significant overall effect of tested material (*p* = 0.0312) and a significant timing × material interaction (*p* < 0.0001). Šídák-adjusted pairwise comparisons showed significantly greater inhibition by the extract than by cinnamaldehyde during virus-compound preincubation (*p* = 0.0376) and during adsorption (*p* = 0.0020), whereas the differences at the other time-of-addition conditions were not significant. For VZV, the overall effect of tested material was not significant (*p* = 0.0655), whereas the timing × material interaction was significant (*p* = 0.0336). Only the virus-compound preincubation condition differed significantly between the extract and cinnamaldehyde after Šídák correction (*p* = 0.0234).

The pronounced antiviral effect observed following post-entry treatment indicates that the extract and cinnamaldehyde primarily interfere with intracellular stages of viral replication. This inhibition may involve disruption of viral genome replication, protein synthesis, virion assembly, or the prevention of secondary rounds of infection. To investigate this possibility, infected cells were treated with 100 µg/mL of the extract or cinnamaldehyde starting after the 2 h viral adsorption period, and intracellular viral infectivity was assessed at 20 or 35 h post-infection, as described in the Section 2. Both *C. cassia* extract and cinnamaldehyde markedly inhibited the production of infectious progeny virus (Figure 5). Across the two sampling times, inhibition was significantly greater for the extract than for cinnamaldehyde for HSV-1 (main effect of tested material, *p* = 0.0010) and VZV (*p* = 0.0033). There was no significant overall effect of sampling time for HSV-1 (*p* = 0.4899) or VZV (*p* = 0.8995). For HSV-1, the time × material interaction was not significant (*p* = 0.2539), whereas for VZV it was significant (*p* = 0.0145). Šídák-adjusted comparisons showed significantly greater inhibition by the extract at both 20 and 35 h post-infection for HSV-1 (*p* = 0.00097 and *p* = 0.0067, respectively) and VZV (*p* = 0.0052 and *p* = 0.0078, respectively).

To examine the effects of the tested materials on viral nucleic acids, Vero cells were infected with HSV-1 or VZV and treated following the 2 h viral adsorption period with 100 µg/mL of either the ethanolic extract or cinnamaldehyde, or with acyclovir at 10 µg/mL. Viral DNA accumulation was analyzed at 24 h post-infection. For transcript analysis, the HSV-1 immediate-early ICP4/RS1 transcript was quantified at 4 h post-infection, whereas the VZV immediate-early ORF63/IE63 transcript was quantified at 14 h post-infection. As shown in Figure 6, all three treatments significantly reduced viral DNA accumulation and the abundance of the respective immediate-early transcripts compared with the corresponding untreated infected controls (Šídák-adjusted *p* < 0.01 for all comparisons). The extract reduced relative viral DNA to approximately 43% of control for HSV-1 and 46% for VZV, while cinnamaldehyde reduced it to approximately 66% and 67%, respectively. The corresponding immediate-early transcript levels were reduced to approximately 53% and 55% by the extract and to approximately 61% and 60% by cinnamaldehyde for HSV-1 and VZV, respectively.

## 4. Discussion

In the present study, we demonstrated that the ethanolic extract of *Cinnamomum cassia* and cinnamaldehyde exhibit marked antiviral activity against the clinically important alphaherpesviruses HSV-1 and VZV in vitro [18]. Both agents inhibited viral replication in a dose-dependent manner while exhibiting relatively low cytotoxicity, resulting in favorable selectivity indices. Nonlinear regression-based analysis showed that the selectivity index of the extract and cinnamaldehyde was similar against HSV-1 (SI 535.6 and 476.9, respectively), whereas the extract showed a substantially higher selectivity index than cinnamaldehyde against VZV (SI 658.8 and 305.0, respectively). These findings suggest that the relative therapeutic window of the crude extract compared with cinnamaldehyde may differ between the two viruses. Compared with acyclovir, the *C. cassia* extract showed a lower selectivity index against HSV-1 (535.6 vs. 775.5) but a higher selectivity index against VZV under the present in vitro assay conditions (658.8 vs. 460.1). These comparisons should be interpreted in the context of the present cell-based assay and do not by themselves establish comparative clinical efficacy. Cinnamon extracts contain multiple phytochemicals (polyphenols, flavonoids, coumarins, procyanidins, and terpenoids) that could potentially exert additive, complementary, or synergistic biological effects. However, because the present study did not characterize the complete chemical composition of the extract or formally evaluate interactions among individual constituents, the contribution of specific compounds and potential synergistic effects remains to be determined. Similar interactions have been described for other medicinal plant extracts, in which the whole extract exhibits greater biological activity than isolated constituents [38,39].

The comparable susceptibility of HSV-1 and VZV to both treatments suggests that these results are consistent with the possibility of interference with conserved intracellular processes, but do not identify the molecular target. HSV and VZV share similar replication strategies, including nuclear DNA replication, expression of conserved immediate-early and early genes, and dependence on highly conserved viral DNA polymerases. Therefore, compounds interfering with these conserved intracellular processes may exhibit broad-spectrum activity against multiple alphaherpesviruses.

These results for the extracts and cinnamaldehyde are consistent with previous studies reporting significant antiviral activity of ethanolic plant extracts and essential oils against various viruses, including poliovirus, coxsackievirus B1, adenovirus type 3, and herpes viruses [40,41,42,43,44].

The results obtained in this study suggest that the antiviral activity of the extract and cinnamaldehyde is unlikely to result from stable interactions with host cell surface receptors or direct inactivation of viral particles. Instead, any interaction occurring before infection appears to be weak and reversible, as antiviral activity was largely lost after the compounds were removed prior to viral challenge. These findings agree with previous reports indicating that certain plant-derived ethanolic extracts exert limited effects on viral adsorption or receptor binding [41,42,43]. However, they differ from studies proposing that many natural antiviral compounds primarily act by directly inactivating virions or preventing viral attachment and penetration into host cells [45,46]. In fact, previous studies have shown that the antiviral effects of both plant ethanolic extracts and essential oils are attributed to multiple complementary mechanisms that interfere with different stages of the viral life cycle. Due to the hydrophobic character of many essential oils constituents, they may associate with viral lipid membranes, resulting in disruption of viral envelope integrity and impairment of viral attachment, fusion, and entry into host cells [47].

In the present study, all treatments markedly reduced the production of infectious progeny viruses within infected cells (Figure 5), indicating that the antiviral activity extends beyond inhibition of viral entry and predominantly targets intracellular events essential for productive viral replication. The sustained reduction in virus production at both time points suggests that the antiviral effect is maintained throughout the replication cycle rather than representing only a transient delay in viral replication.

The marked reduction in progeny virus production is consistent with inhibition of intracellular viral replication. Also, reduced viral DNA accumulation and reduced abundance of the specific immediate-early transcripts examined were associated with this effect. Although these nucleic-acid changes did not fully account for the pronounced reduction in infectious virus production (Figure 5), the findings suggest that additional intracellular processes, including viral protein expression, genome replication, virion assembly, maturation, or egress, may also be affected. These observations support the concept that cinnamon-derived compounds exert multitarget antiviral activity, a property that may reduce the likelihood of antiviral resistance. If confirmed, compounds acting independently of viral thymidine kinase may retain activity against acyclovir-resistant herpesvirus isolates.

It is worth noting that the decrease in the specific immediate-early transcript abundance observed after treatment with the extract or cinnamaldehyde could be a concomitant result of interference with other intracellular replication processes rather than a direct effect on transcription itself.

The identification of agents that interfere with intracellular stages of herpesvirus replication is particularly relevant in the context of recent efforts to develop antiviral drugs with mechanisms distinct from those of conventional nucleoside analogues. Current research on HSV therapeutics has increasingly focused on alternative viral and host targets, including the helicase–primase complex and other processes required for productive viral replication. In this context, the post-entry activity observed for both *C. cassia* extract and *cinnamaldehyde* is of interest because it suggests that their antiviral effects are not limited to interference with viral attachment or direct virion inactivation. Although the precise molecular target was not identified in the present study, the time-of-addition experiments together with reduced viral DNA accumulation and reduced abundance of the specific immediate-early transcripts examined support interference with intracellular events occurring after viral entry. These findings are consistent with interference at one or more intracellular stages of the viral replication cycle. Further studies should therefore determine whether these effects involve viral DNA replication, regulation of viral gene expression, protein synthesis, virion assembly, or modulation of host pathways required for efficient HSV and VZV replication. Possible mechanisms include inhibition of viral DNA polymerase activity, suppression of immediate-early viral gene expression, modulation of host signaling pathways required for efficient viral replication, or impairment of viral protein synthesis. Alternatively, cinnamaldehyde may influence host–cell signaling pathways, including NF-κB and MAPK signaling, that contribute to efficient herpesvirus replication. These possibilities warrant future investigation using viral protein expression analyses, transcriptomic approaches, and enzymatic assays.

Collectively, these findings support the growing potential of ethanolic extracts and essential oils as multifunctional antiviral agents and underscore the need for further studies to elucidate their molecular targets and optimize their integration into antiviral treatment strategies. In addition, isolating and purifying the bioactive antiviral constituent(s) of the extract may enhance its antiviral efficacy and improve its therapeutic potential against herpesvirus infections.

Finally, the present study has several limitations. First, all experiments were performed in Vero cells, which do not fully recapitulate the complexity of human epithelial or neuronal infection. Future studies should therefore include primary human fibroblasts and keratinocytes, which more closely resemble the natural target cells of HSV and VZV infection. Second, only in vitro antiviral activity was evaluated, and pharmacokinetic properties, bioavailability, and systemic toxicity remain unknown. Third, the precise molecular targets responsible for antiviral activity were not identified. Although the HSV-1 ICP4/RS1 and VZV ORF63/IE63 transcripts were examined at 4 h and 14 h post-infection, respectively, only one immediate-early transcript was analyzed for each virus. Therefore, these data do not establish a generalized inhibition of viral transcription and do not identify the precise intracellular target of the tested materials. The observed reduction in transcript abundance may reflect a direct or indirect consequence of interference with one or more intracellular replication processes. Future kinetic studies examining representative immediate-early, early, and late genes at multiple time points will be required to determine more precisely which stage(s) of the viral replication cycle are affected. Fourth, the dried cinnamon bark was obtained as a commercial product marketed as *C. cassia* and was not taxonomically authenticated through a certified botanical supplier or voucher specimen. Consequently, its exact botanical identity cannot be independently verified. The findings obtained with the crude extract should therefore be interpreted as specific to the commercial preparation tested and should not be generalized without qualification to taxonomically authenticated *C. cassia* material. Future studies should use voucher-documented plant material together with chromatographic profiling and quantitative standardization. Finally, the ethanolic extract used in the present study was not chemically profiled or standardized. Therefore, the abundance of cinnamaldehyde in the tested extract and the identity and contribution of other constituents cannot be determined from the present data. The antiviral findings obtained with the crude extract should consequently be regarded as specific to the preparation tested. Future studies should include chromatographic profiling and quantitative standardization of the extract before mechanistic attribution to individual constituents.

## 5. Conclusions

The present study shows that the ethanolic extract prepared from commercial cinnamon bark marketed as Cinnamomum cassia and cinnamaldehyde exhibit in vitro antiviral activity against HSV-1 and VZV in Vero cells. Both treatments inhibited viral replication in a dose-dependent manner and reduced the production of infectious progeny viruses. The time-of-addition experiments further suggest that their antiviral effects are mainly associated with events occurring after viral entry, rather than with direct inactivation of viral particles or inhibition of viral attachment.

Treatment of infected cells was also associated with reduced viral DNA accumulation and reduced abundance of the specific immediate-early transcripts examined. However, the present experiments do not establish the precise molecular target(s) responsible for these effects, and it remains unclear which specific intracellular step(s) of the viral replication cycle are affected.

Overall, these findings provide preliminary evidence that *C. cassia* extract and cinnamaldehyde may interfere with HSV-1 and VZV replication in vitro. Further studies using additional relevant cell models, chemically characterized preparations, and in vivo systems will be required to define their molecular mechanisms, evaluate their antiviral efficacy and safety, and determine their potential relevance as candidates for future antiviral development.

## Data Availability

Data are contained within the article.
